# Association of Sociodemographic Factors With Immunotherapy Receipt for Metastatic Melanoma in the US

**DOI:** 10.1001/jamanetworkopen.2020.15656

**Published:** 2020-09-02

**Authors:** Justin T. Moyers, Amie Patel, Wendy Shih, Gayathri Nagaraj

**Affiliations:** 1 Division of Hematology and Oncology, Department of Internal Medicine, Loma Linda University, Loma Linda, California; 2 Department of Internal Medicine, Loma Linda University, Loma Linda, California; 3 School of Public Heath, Loma Linda University, Loma Linda, California

## Abstract

**Importance:**

Strides to improve survival in metastatic melanoma have been made with the use of immunotherapeutic agents in the form of immune checkpoint inhibitors.

**Objective:**

To examine the factors associated with immunotherapy receipt in patients with metastatic melanoma in the era of immune checkpoint inhibitors and the Patient Protection and Affordable Care Act.

**Design, Setting, and Participants:**

This cohort study used data on 9882 patients with metastatic melanoma diagnosed from January 1, 2013, to December 31, 2016, from the National Cancer Database. Patients who did not have documentation regarding immunotherapy receipt were excluded. Data analysis was performed from July 1, 2019, to December 15, 2019.

**Exposure:**

Receipt of immunotherapy.

**Main Outcomes and Measures:**

The primary outcome was the association of receipt of immunotherapy as first-line therapy with sociodemographic factors. The secondary outcome was overall survival by receipt of immunotherapy.

**Results:**

A total of 9512 patients (mean [SD] age, 65.1 [14.4] years; 6481 [68.1%] male; 9217 [96.9%] White) met the criteria for treatment analysis. A total of 3428 (36.0%) received immunotherapy, and 6084 (64.0%) did not. Increasing age (odds ratio [OR], 0.98; 95% CI, 0.97-0.98; *P* < .001) and increasing Charlson-Deyo comorbidity index (OR, 0.86; 95% CI, 0.80-0.92; *P* < .001) were associated with lower odds of receiving immunotherapy on regression analysis. Diagnosis in Medicaid expansion states (OR, 1.16; 95% CI, 1.05-1.27; *P* = .003), treatment at an academic or integrated cancer network program (OR, 1.59; 95% CI, 1.45-1.75; *P* < .001), and residence within the highest quartile of high school graduation rate zip code area (OR, 1.31; 95% CI, 1.09-1.56; *P* = .003) were associated with an increased likelihood of receiving immunotherapy. Median overall survival was 10.1 months (95% CI, 9.6-10.6 months) among all patients. Patients who received first-line immunotherapy had a median overall survival of 18.4 months (95% CI, 16.6-20.1 months) compared with 7.5 months (95% CI, 7.0-7.9 months) (*P* < .001) among patients who did not.

**Conclusions and Relevance:**

In this cohort study, patients who received immunotherapy for metastatic melanoma had improved overall survival. Residence in Medicaid expansion states, younger age, low comorbidity index, care at academic medical centers or integrated network cancer programs, and residence in zip codes within the highest quartile of high school graduation were associated with an increased likelihood of receiving immunotherapy. Recognizing sociodemographic associations with treatment receipt is important to identify potential barriers to treatment.

## Key Points

**Question:**

Are sociodemographic factors associated with likelihood of receiving immunotherapy for patients diagnosed with metastatic melanoma in the US?

**Findings:**

In this cohort study of 9512 metastatic melanoma cases diagnosed between 2013 and 2016 in the National Cancer Database, factors associated with receiving immunotherapy included diagnosis in Medicaid expansion states, residence in areas with high rates of high school graduation, and treatment at academic cancer centers or integrated cancer networks.

**Meaning:**

This study found that patients with metastatic melanoma diagnosed in Medicaid expansion states, treated at academic or integrated cancer centers, and living in high graduation–rate areas were more likely to receive immunotherapy.

## Teaser

This cohort study examines the sociodemographic factors associated with immunotherapy receipt in patients with metastatic melanoma in the era of immune checkpoint inhibitors and the Patient Protection and Affordable Care Act in the US.

## Introduction

The mainstay treatment for unresectable or metastatic melanoma is systemic therapy. Before the introduction of immune checkpoint inhibitors (ICIs), the standard of care for most patients with metastatic melanoma was dacarbazine administered as single-agent therapy or part of combination therapy.^1,2^ Immunotherapy with high-dose interleukin 2 showed durable response in a few patients but was associated with significant toxic effects^2,3^; however, immunotherapeutic agents in the form of ICIs, including ipilimumab, nivolumab, and pembrolizumab, have substantially improved outcomes and become the standard of care for metastatic melanoma during the past decade.^4^

After the landmark study of the cytotoxic T-lymphocyte–associated protein 4 inhibitor ipilimumab by Hodi et al,^5^ ipilimumab became the first ICI approved for metastatic melanoma in 2011.^6^ Subsequently, single-agent anti–programmed cell death protein 1 agents have shown superior efficacy compared with ipilimumab, including pembrolizumab in KEYNOTE-006 (Study to Evaluate the Safety and Efficacy of Two Different Dosing Schedules of Pembrolizumab [MK-3475] Compared to Ipilimumab in Participants With Advanced Melanoma)^7,8,9^ and nivolumab in CHECKMATE-066 (Study of Nivolumab [BMS-936558] Compared With Dacarbazine in Untreated, Unresectable, or Metastatic Melanoma).^10,11^ Combination therapy with nivolumab and ipilimumab also showed improved overall survival,^12,13,14^ leading to approval of the combination in the first-line setting.^11,15^

Despite the improvement in outcomes with ICIs for metastatic melanoma, previous studies^16,17^ have found that sociodemographic health disparities are associated with limited access to effective therapies for melanoma, including immunotherapy. Underinsurance may be associated with delays in surgery and treatment at low-volume centers. Insurance status is also associated with stage of diagnosis, with uninsured patients presenting at more advanced disease stages compared with privately insured patients.^18^ Use of systemic therapy has been administered more frequently to those who were living in high-income areas, younger individuals, and married individuals.^19^

**Figure 1.**
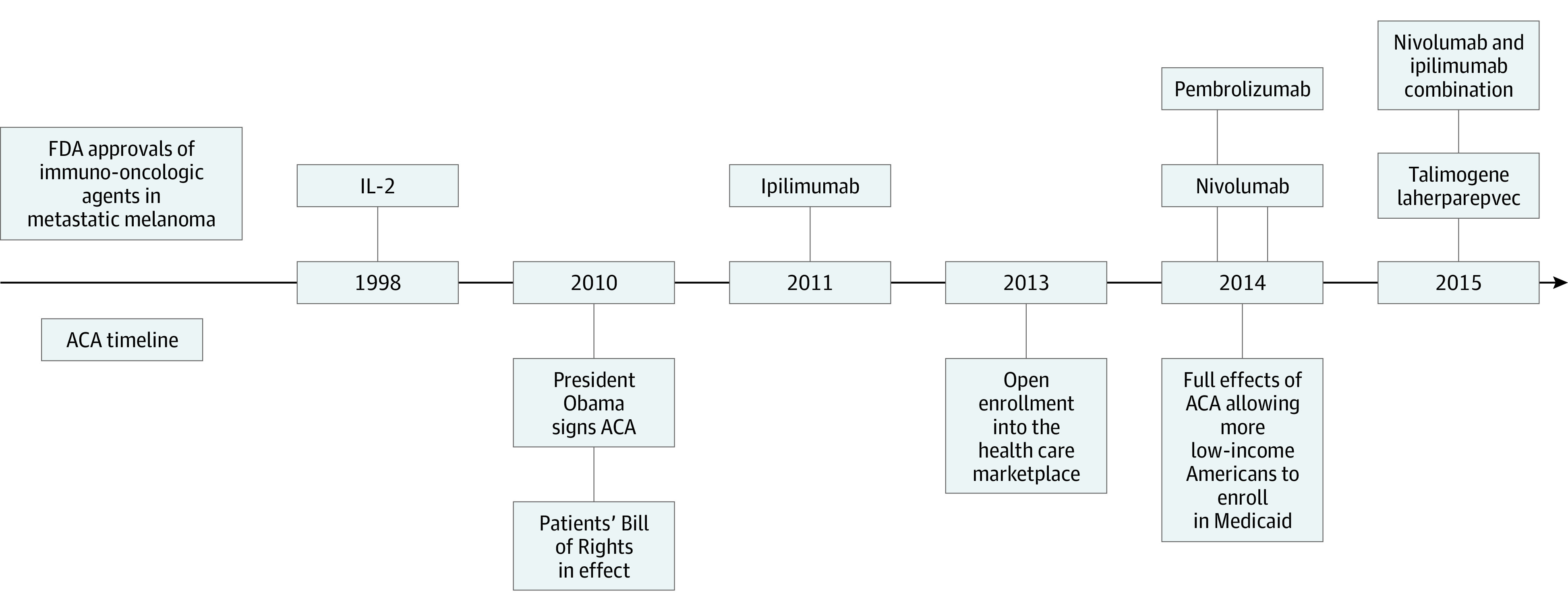
Key Dates in US Food and Drug Administration (FDA) Approvals of Immuno-oncologic Agents for Metastatic Melanoma and the Enactment of the Patient Protection and Affordable Care Act (ACA) Data have been reported elsewhere.^6,9,11,15,24^ IL indicates interleukin.

Recently, the Patient Protection and Affordable Care Act (ACA) has included policies to reduce the socioeconomic and racial/ethnic disparities in health care access. When President Obama signed the ACA in 2010, a new patient’s bill of rights was implemented to protect individuals with preexisting conditions, improve choice of physicians, and end insurance limits on care. This new bill of rights has allowed many patients with a new cancer diagnosis to gain and keep coverage.^20^ The Medicaid expansion provision of the ACA encouraged states to expand Medicaid coverage by covering 100% of the expansion in the first 3 years and 90% afterward.^21^ In 2013, the Medicaid expansion open enrollment period for states commenced, and by the beginning of 2017, 32 states had implemented expansion (eFigure 1 in Supplement). ^22^ In addition, the ACA established insurance marketplaces that provide access to more affordable private insurance plans and government subsidies.^23^ Key dates for implementation of ACA and approval of immunotherapeutic agents are summarized in Figure 1.

Since enactment of the ACA, an estimated 20 million people have gained health insurance coverage through Medicaid expansion and health care insurance marketplaces.^25^ The uninsured rate of the population younger than 65 years in the US decreased from 17% to 10% between 2013 and 2016, and the uninsured rate reduction in the Medicaid expansion states was 50% compared with 31% in the nonexpansion states.^26^ With improved access to health care, patients with metastatic melanoma may have increased opportunity for treatment with immunotherapy.

The 2016 update of the National Cancer Database (NCDB) participant user file added a new code for Medicaid expansion grouped by each state’s Medicaid expansion status. In addition, updated demographic data based on the 5-year aggregate of the US Census Bureau’s 2012-2016 American Community Survey were included.^27^ Therefore, we used the updated 2016 NCDB participant user file in the time since ACA enactment to evaluate the association of sociodemographic health factors, including state Medicaid expansion status, with receipt of immunotherapy for metastatic melanoma.

## Methods

### Data Source

This cohort study used data from the NCDB, which was established in 1989 and is a nationwide, facility-based, comprehensive clinical surveillance resource oncology data set that currently captures 52% of all melanoma cases and 72% of all newly diagnosed malignant tumors in the US annually.^28^ The NCDB is a joint project of the American Cancer Society and the Commission on Cancer of the American College of Surgeons. By waiver of determination, Loma Linda University did not require institutional review board approval of this study. The data were deidentified by the NCDB. This study followed the Strengthening the Reporting of Observational Studies in Epidemiology (STROBE) reporting guideline.

### Study Population

This retrospective cohort study used melanoma cases registered in the NCDB that were diagnosed from January 1, 2013, to December 31, 2016, with follow-up through 2018. Data were analyzed from July 1, 2019, to December 15, 2019. We queried the database to identify patients with stage IV melanoma, also referred to as metastatic melanoma in this article. The primary outcome was the association of receipt of immunotherapy as first-line therapy with sociodemographic factors. Patients were divided into groups that received immunotherapy or that did not receive immunotherapy; cases without documentation of either were excluded. The secondary outcome was overall survival in the group that received immunotherapy vs the group that did not receive this therapy. Cases diagnosed in 2016 do not have survival data recorded and could not be included in the survival analysis.

### Statistical Analysis

Covariates examined in the treatment groups included age, sex, year of diagnosis, Charlson-Deyo comorbidity index, primary payer, state Medicaid expansion status, treatment region, metropolitan vs suburban residence, facility type, educational level, and income level. Community type was categorized as metropolitan for facilities located in counties with populations of 250 000 or greater and suburban for facilities in counties with populations less than 250 000. Medicaid expansion status was defined by the state of residence at the time of cancer diagnosis and by categorization into Medicaid expansion at any time from 2013 to 2016, Medicaid nonexpansion, or suppressed for ages younger than 40 years. Educational and income levels were classified by the 5-year estimates of the 2012 to 2016 update of the US Census Bureau’s American Community Survey results. Educational level was defined by the percentage quartile of high school graduates and median income by the quartile range of income. The primary payer as registered at the time of diagnosis was used to determine insurance status. The eAppendix in the Supplement includes a complete listing of variables used in the analysis.

Continuous variables are presented as means (SDs). Categorical variables are presented as number (percentage), with comparisons performed by χ^2^ analysis. Survival was calculated from time of diagnosis to time to last contact or death in months. Survival analyses were performed using the Kaplan-Meier method and log-rank test. Logistic regression was used to examine factors associated with immunotherapy receipt. A 2-sided *P* < .05 indicated statistical significance.

 All statistical analyses were performed using SPSS statistical software, version 25 (IBM Inc); R, version 3.6.2 (R Foundation for Statistical Computing); and Microsoft Excel (Microsoft Corp).

## Results

### Baseline Characteristics of the Data Sample

Among 583 212 melanoma diagnoses in the database, 9882 stage IV diagnoses were identified, with 7027 available for survival analysis and 9512 meeting the criteria for treatment analysis (mean [SD] age, 65.1 [14.4] years; 6481 [68.1%] male; 9217 [96.9%] White). A flow diagram detailing patient allotment is shown in eFigure 2 in the Supplement. A total of 3428 patients (36.0%) received immunotherapy, and 6084 (64.0%) did not. The Table summarizes demographic data stratified by treatment cohort. Immunotherapy receipt by year of diagnosis increased yearly from 23.6% receiving immunotherapy in 2013 to 48.0% receiving immunotherapy in 2016 (eFigure 3 in the Supplement).

**Table.** Baseline Characteristics of the Study Population by Immunotherapy Receipt^a^

Characteristic	Total population (N = 9512)	Immunotherapy		*P* value
		No (n = 6084)	Yes (n = 3428)	
Age, mean (SD), y	65.1 (14.4)	64.6 (14.4)	62.6 (14.1)	<.001
Race				
White	9217 (96.9)	5895 (64.0)	3322 (36.0)	>.99
Black	132 (1.4)	85 (64.4)	47 (35.6)	
Unknown	163 (3.1)	104 (63.8)	59 (36.2)	
Sex				
Male	6481 (68.1)	4128 (63.7)	2353 (36.3)	.63
Female	3031 (31.9)	1956 (64.5)	1075 (35.5)	
Charlson-Deyo comorbidity index				
0	7172 (75.4)	4435 (61.8)	2737 (38.2)	<.001
1	1565 (16.5)	1094 (69.9)	471 (30.1)	
2	492 (5.2)	350 (71.1)	142 (28.9)	
≥3	283 (3.0)	205 (72.4)	78 (27.6)	
Facility type				
Community cancer program	745 (8.3)	540 (72.5)	205 (27.5)	<.001
Comprehensive community cancer program	3377 (37.6)	2386 (70.7)	991 (29.3)	
Academic or research program	3684 (41.0)	2106 (57.2)	1578 (42.8)	
Integrated network cancer program	1184 (13.2)	778 (65.7)	406 (34.3)	
Primary payer				
Not insured	375 (3.9)	276 (73.6)	99 (26.4)	<.001
Private insurance or managed care	3343 (35.1)	1915 (57.3)	1428 (42.7)	
Medicaid	721 (7.6)	486 (67.4)	235 (32.6)	
Medicare	4722 (49.6)	3184 (67.4)	1538 (32.6)	
Other government	166 (1.7)	103 (62.0)	63 (38.0)	
Insurance status unknown	185 (1.9)	120. (64.9)	65 (35.1)	
Facility location^b^				
New England	464 (5.2)	279 (60.1)	195 (39.9)	<.001
Mid-Atlantic	1261 (14.0)	762 (60.4)	499 (39.6)	
South Atlantic	2083 (23.2)	1379 (66.2)	704 (33.8)	
East North Central	1466 (16.3)	935 (63.8)	531 (36.2)	
East South Central	663 (7.4)	353 (70.0)	199 (30.0)	
West North Central	685 (7.6)	407 (59.4)	278 (40.6)	
West South Central	675 (7.5)	490 (72.6)	185 (27.4)	
Mountain	526 (5.9)	312 (59.3)	214 (40.7)	
Pacific	1167 (13.0)	782 (67.0)	385 (33.0)	
Income, median quartiles 2012-2016, $				
<40 227	1358 (14.5)	935 (68.9)	423 (31.1)	<.001
40 227-50 353	2099 (22.4)	1367 (65.1)	732 (34.9)	
50 354-63 332	2331 (24.8)	1468 (63.0)	863 (37.0)	
≥63 333	3603 (38.4)	227 (61.8)	1376 (38.2)	
No high school degree, quartiles 2012-2016, %				
≥17.6	1555 (16.5)	1076 (69.2)	479 (30.8)	<.001
10.9-17.5	2388 (25.4)	1546 (64.7)	842 (35.3)	
6.3-10.8	2866 (30.5)	1808 (63.1)	1058 (36.9)	
<6.3	2602 (27.6)	1580 (60.7)	1022 (39.3)	
Metropolitan vs suburban 2013 categorization^c^				
Metropolitan areas	7682 (82.8)	4857 (63.2)	2825 (36.8)	.002
Suburban areas	1390 (14.9)	1069 (67.3)	519 (32.7)	
State Medicaid expansion status				
Expansion	5419 (57.0)	3401 (62.8)	2018 (37.2)	<.001
Nonexpansion	3571 (37.5)	2409 (67.5)	1162 (32.5)	
Unknown	522 (5.5)	1069 (52.5)	519 (47.5)	

^a^ Data are presented as number (percentage) of patients unless otherwise indicated.

^b^ New England includes Connecticut, Massachusetts, Maine, New Hampshire, Rhode Island, and Vermont; Mid-Atlantic, New Jersey, New York, and Pennsylvania; South Atlantic, District of Columbia, Delaware, Florida, Georgia, Maryland, North Carolina, South Carolina, Virginia, and West Virginia; East North Central, Illinois, Indiana, Michigan, Ohio, and Wisconsin; East South Central, Alabama, Kentucky, Mississippi, and Tennessee; West North Central, Iowa, Kansas, Minnesota, Missouri, North Dakota, Nebraska, and South Dakota; West South Central, Arkansas, Louisiana, Oklahoma, and Texas; Mountain, Arizona, Colorado, Idaho, Montana, New Mexico, Nevada, Utah, and Wyoming; Pacific, Alaska, California, Hawaii, Oregon, and Washington.

^c^ Metropolitan was defined as counties with a population of 250 000 or greater; suburban, 250 000 population or less.

### Survival and Time-to-Event Analysis

**Figure 2.**
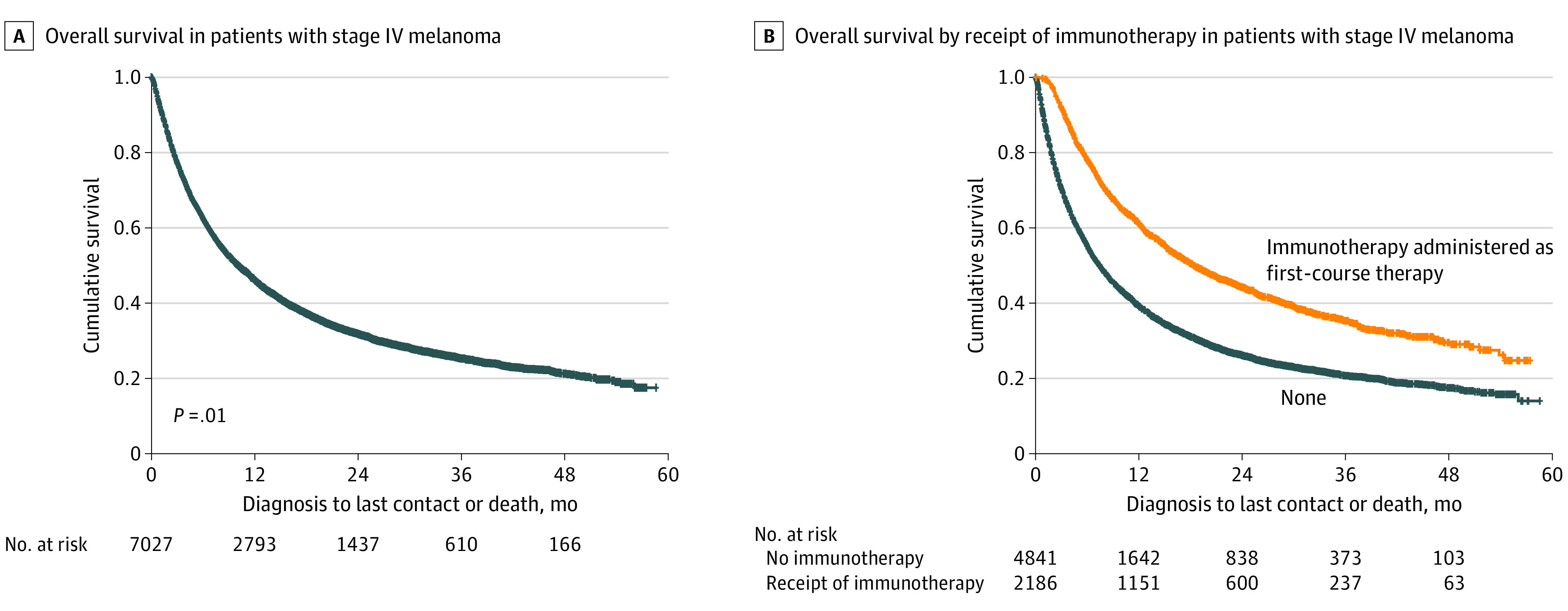
Overall Survival Among All Patients With Stage IV Melanoma and by Receipt of Immunotherapy Data are from the National Cancer Database, 2013 to 2015.

Median overall survival among all patients regardless of treatment was 10.1 months (95% CI, 9.6-10.6 months) (Figure 2A). Improved median overall survival was observed for patients who received immunotherapy as first-line therapy compared with patients who did not (18.4 months [95% CI, 16.6-20.1] vs 7.5 months [95% CI, 7.0-7.9]; *P* < .001) (Figure 2B). Median time to immunotherapy was 54.0 days (IQR, 34.0-84.0 days) for patients diagnosed in nonexpansion states vs 52.0 days (IQR, 34.0-86.0 days) in those diagnosed in expansion states (*P* = .50) (eFigure 4 in the Supplement).

### Logistic Regression

**Figure 3.**
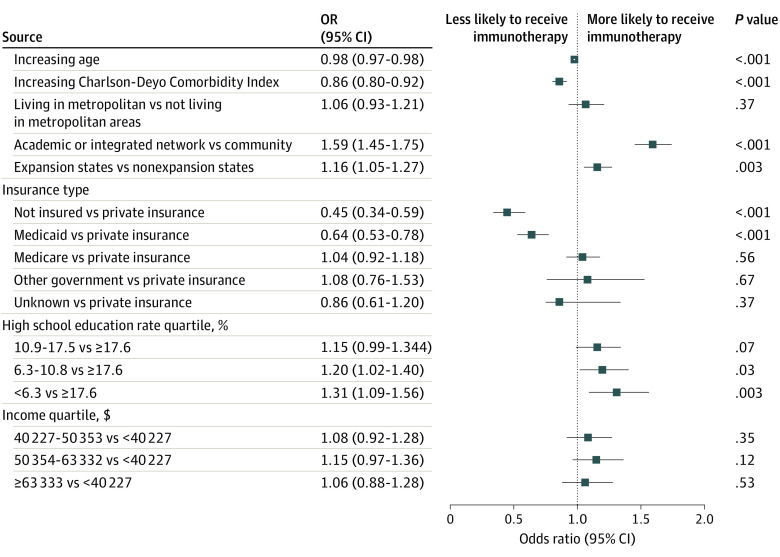
Forest Plot of Regression Analysis for Receipt of Immunotherapy by Demographic Factors

Increasing age (OR, 0.98; 95% CI, 0.97-0.98; *P* < .001) and increasing Charlson-Deyo comorbidity index (OR, 0.86; 95% CI, 0.80-0.92; *P* < .001) were associated with decreased odds of receiving immunotherapy (Figure 3). Diagnosis in Medicaid expansion states was associated with improved likelihood of receiving immunotherapy (OR, 1.16; 95% CI, 1.05-1.27; *P* = .003). Compared with having private insurance, the uninsured patients (OR, 0.45; 95% CI, 0.34-6 0.59; *P* < .001) or patients with Medicaid (OR, 0.64; 95% CI, 0.53-0.78; *P* = .003) were less likely to receive immunotherapy, whereas no statistically significant effect was noted for payer types of Medicare (OR, 1.04; 95% CI, 0.92-1.18; *P* = .56), other government (OR, 1.08; 95% CI, 0.76-1.53; *P* = .67), and unknown payer (OR, 0.86; 95% CI, 0.61-1.20; *P* = .37). Patients treated at academic cancer centers and integrated cancer networks were more likely to receive immunotherapy compared with those in community centers (OR, 1.59; 95% CI, 1.45-1.75; *P* < .001). Patients residing in zip codes with the highest quartile of high school graduation (<6.3% no high school degree) compared with the lowest quartile of completion (≥17.6% no high school degree) had a significantly increased likelihood of receiving immunotherapy (OR, 1.31; 95% CI, 1.09-1.56; *P* = .003), whereas the remaining quartiles were 10.9% to 17.5% (OR, 1.15; 95% CI, 0.99-1.34; *P* = .07) and 6.3% to 10.8% (OR, 1.20; 95% CI, 1.02-1.40; *P* = .03). Compared with the lowest income quartile (<$40 227), the upper 3 quartiles of income of $40 227 to $50 353 (OR, 1.08; 95% CI, 0.92-1.28; *P* = .35), $50 354 to $63 332 (OR, 1.15; 95% CI, 0.97-1.36; *P* = .12), and $63 333 or greater (OR, 1.06; 95% CI, 0.88-1.28; *P* = .54) had no association with receipt of immunotherapy.

## Discussion

Previous studies^29,30,31,32,33^ have analyzed the NCDB cohort before our analysis of the 2016 participant user file. These analyses evaluated the disparity in use of immunotherapy for all stages of melanoma in diagnosis years before 2013,^29^ before 2014,^30,31^ and in 2015^32^ and for stage III disease.^33^ These prior studies^29,30,31,32,33^ indicated the presence of comorbidities, older age, government or no insurance, lower educational and income levels, and treatment at a community practice as factors associated with decreased receipt of immunotherapy. Our study further supports that many of these prior demographic factors may be associated with receipt of immunotherapy with the addition of cases diagnosed in 2015 and 2016. Of importance, the present study showed that state Medicaid expansion status may be an additional factor associated with receipt of immunotherapy.

The ACA has had an effect on multiple aspects of cancer care. An analysis by Han et al^34^ of the National Cancer Institute’s Surveillance, Epidemiology, and End Results (SEER) database showed a decrease in uninsured patients with new cancer diagnoses after the first year of Medicaid expansion among many common cancer types and demographic groups. A decreased disparity in the uninsured population was observed when compared by race and poverty level. A separate analysis of SEER data after implementation of ACA by Chino et al^35^ showed a decrease in uninsured status in Medicaid expansion states for patients receiving radiotherapy for oncologic indications. A recent study of lung, breast, and colon cancers in the NCDB by Takvorian et al^36^ found an increase in insured status without significant change to timeliness of treatment. A recent study by Garland et al^37^ that compared Mohs access for melanoma in situ and rare cutaneous tumors before and after ACA implementation did not find increased access to this expensive surgical procedure in the SEER database. The present study adds to the increasing literature indicating that sociodemographic disparities are associated with patients’ access to health care. Specifically, this study of NCDB data is the first, to our knowledge, to find an increased likelihood of receiving a specific category of treatment (immunotherapy) in patients diagnosed with stage IV melanoma in Medicaid expansion states.

The strongest association for receiving immunotherapy in the present study was among patients receiving care at academic or integrated cancer networks compared with community programs. This finding echoes a recent study by Carey et al^38^ that reported improved survival in patients with head and neck cancer treated at academic comprehensive cancer programs and integrated network cancer programs compared with community cancer centers. Care at academic centers may allow for access to experts well versed in treating patients with newer therapies and managing their complications.^39^

Comparisons of real-world outcomes vs clinical trials have found similarity between outcomes in control arms^40^ and experimental treatments.^41^ Median overall survival among treatment-naive patients with metastatic and advanced melanoma reported in immunotherapy trials were 15.9 to 19.9 months for ipilimumab,^7,13,14^ 36.9 to 37.5 months for nivolumab,^14,42^ 32.7 months for pembrolizumab,^7^ and more than 60 months for the combination of nivolumab and ipilimumab.^14^ Although all 4 of these treatment regimens were approved for use during this study’s timeframe, surveys on community practice treatment trends favored ipilimumab use with subsequent adoption of the other regimens in the community.^43^ The median overall survival of 18.4 months in this study is similar to that associated with ipilimumab treatment in the aforementioned trials but less than that of single-agent anti–programmed cell death protein 1 or combination checkpoint inhibition. Our finding further supports increasing evidence that real-world treatment outcomes are similar to those in clinical trials as has already been seen in non–small cell lung cancer.^44^

### Limitations

This study has limitations. The NCDB is a retrospective database that does not cover the entire population. Furthermore, only first-line systemic therapy is recorded. Immunotherapy given as second-line treatment may be associated with improved survival,^45^ but receipt would not be recorded in the database. Because the insurance status and Medicaid expansion status are suppressed for patients younger than 40 years and cannot be evaluated, generalizability of results to patients younger than 40 years cannot be made. The specific agent or agents used as immunotherapy are not recorded and could include ICIs, vaccine therapy, interleukins, or other biologic response-modifying agents. In addition, disease-specific survival is not recorded.^46^ The data used in the study are derived from a deidentified NCDB file.

## Conclusions

Using the NCDB, we found that immunotherapy use for metastatic melanoma has increased since approval of ICIs by the US Food and Drug Administration. Nearly 50% of patients with metastatic melanoma were receiving first-line immunotherapy in 2016. An improvement in survival by more than 10 months was found in the population of the NCDB who received immunotherapy. Furthermore, patients with residence in Medicaid expansion states, younger age, low Charlson-Deyo comorbidity index, treatment at academic medical centers or integrated network cancer programs, and zip codes within the highest quartile of high school graduation were more likely to receive immunotherapy.

## Notes

Supplement. **eAppendix.** Variable Dictionary **eFigure 1.** Illustrative Map of the United States of America Recording Expansion States as of December 2016 as Categorized by NCDB in Our Study **eFigure 2.** CONSORT Diagram Listing Patient Allotment in Analysis **eFigure 3.** Immunotherapy Receipt as Percentage of Total Population by Year **eFigure 4. **Time From Diagnosis to Treatment With Immunotherapy in Days Separated by State Medicaid Expansion Status **eTable.** Payer Status at Time of Diagnosis by Medicaid Expansion Status
