# Supplementary material for: Association of Sociodemographic Factors With Immunotherapy Receipt for Metastatic Melanoma in the US
Source: JAMA Netw Open. 2020 Sep 2;3(9):e2015656. doi: 10.1001/jamanetworkopen.2020.15656 (PMC7489862; doi:10.1001/jamanetworkopen.2020.15656)
Supplement: Supplement. — eAppendix. Variable Dictionary eFigure 1. Illustrative Map of the United States of America Recording Expansion States as of December 2016 as Categorized by NCDB in Our Study eFigure 2. CONSORT Diagram Listing Patient Allotment in Analysis eFigure 3. Immunotherapy Receipt as Percentage of Total Population by Year eFigure 4. Time From Diagnosis to Treatment With Immunotherapy in Days Separated by State Medicaid Expansion Status eTable. Payer Status at Time of Diagnosis by Medicaid Expansion Status [file jamanetwopen-e2015656-s001.pdf]

Supplementary Online Content

Moyers JT, Patel A, Shih W, Nagaraj G. Association of sociodemographic factors with immunotherapy receipt for metastatic melanoma in the US. *JAMA Netw Open*. 2020;3(9):e2015656.
doi:10.1001/jamanetworkopen.2020.15656

eAppendix. Variable Dictionary

eFigure 1. Illustrative Map of the United States of America Recording Expansion States as of December 2016 as Categorized by NCDB in Our Study

eFigure 2. CONSORT Diagram Listing Patient Allotment in Analysis

eFigure 3. Immunotherapy Receipt as Percentage of Total Population by Year

eFigure 4. Time From Diagnosis to Treatment With Immunotherapy in Days Separated by State Medicaid Expansion Status

eTable. Payer Status at Time of Diagnosis by Medicaid Expansion Status

This supplementary material has been provided by the authors to give readers additional information about their work.

© 2020 Moyers JT et al. *JAMA Network Open.*

eAppendix. Variable Dictionary
Listing of Variables used in Analysis from NCDB PUF Data Dictionary

**Manuscript Variable:**	**PUF Data Item and Options**
Facility Type	FACILITY_TYPE_CD Facility Type<br>1. Community Cancer Program<br>2. Comprehensive Community Cancer Program<br>3. Academic/Research Program (includes NCI-designated comprehensive cancer centers)<br>4. Integrated Network Cancer Program
Region	FACILITY_LOCATION_CD<br>1 New England<br>2 Middle Atlantic<br>3 South Atlantic<br>4 East North Central<br>5 East South Central<br>6 West North Central<br>7 West South Central<br>8 Mountain<br>9 Pacific
Age at Diagnosis	AGE
Sex	Sex<br>1 Male<br>2 Female
Race	RACE
Insurance Status	INSURANCE_STATUS<br>0-Not Insured<br>1-Private Insurance / Managed Care<br>2-Medicaid<br>3-Medicare<br>4-Other Government<br>9-Insurance Status Unknown
Education	NO_HSD_QUAR_2016<br>1 17.6% or more<br>2 10.9% - 17.5%<br>3 6.3% - 10.8%<br>4 Less than 6.3%
Income Level	Med_inc_quar_2016<br>1 Less than $38,000<br>2 $38,000 - $47,999<br>3 $48,000 - $62,999<br>4 $63,000 +
Urban Rural	UR_CD_13<br>1 Counties in metro areas of 1 million population or more<br>2 Counties in metro areas of 250,000 to 1 million population<br>3 Counties in metro areas of fewer than 250,000 population<br>4 Urban population of 20,000 or more, adjacent to a metro area.<br>5 Urban population of 20,000 or more, not adjacent to a metro area.<br>6 Urban population of 2,500 to 19,999, adjacent to a metro area.

© 2020 Moyers JT et al. *JAMA Network Open.*

	7 Urban population of 2,500 to 19,999, not adjacent to a metro area.<br>8 Completely rural or less than 2,500 urban population, adjacent to a metro area<br>9 Completely rural or less than 2,500 urban population, not adjacent to a metro area
Medicaid Expansion Status	MEDICAID_EXPN_CODE<br>0-Non-Expansion States<br>1-January 2014 Expansion States<br>2-Early Expansion States (2010- 2013)<br>3-Late Expansion States (after Jan. 2014)<br>9-Suppressed for Ages 0-39
CDCI	CDCC_TOTAL_BEST<br>0 Total Charlson Deyo Score of 0<br>1 Total Charlson-Deyo score of 1<br>2 Total Charlson-Deyo Score of 2<br>3 Total Charlson –Deyo Score of 3 or more
Year	YEAR_OF_DIAGNOSIS<br>Four Digit Year
Stage	ANALYTIC_STAGE_GROUP<br>0 Stage 0<br>1 Stage I<br>2 Stage II<br>3 Stage III<br>4 Stage IV<br>5 Occult (lung only)<br>8 AJCC Staging not applicable<br>9 AJCC Stage group unknown
Immunotherapy	RX_SUMM_IMMUNOTHERAPY<br>00 None, immunotherapy was not part of the planned first course of therapy.<br>01 Immunotherapy administered as first course therapy.<br>82 Immunotherapy was not recommended/administered because it was contraindicated due to patient risk factors (ie, comorbid conditions, advanced age).<br>85 Immunotherapy was not administered because the patient died prior to planned or recommended therapy.<br>86 Immunotherapy was not administered. It was recommended by the patient's physician, but was not administered as part of the first course of therapy. No reason was stated in patient record.<br>88 Immunotherapy was recommended, but it is unknown if it was administered.<br>99 It is unknown whether a immunotherapeutic agent(s) was recommended or administered because it is not stated in patient record. Death certificate only.
Vital Status	PUF_VITAL_STATUS<br>0- Dead<br>1- Alive
Survival Time	DX_LASTCONTACT_DEATH_MONTHS<br>“0.001– 8887.9”

© 2020 Moyers JT et al. *JAMA Network Open.*

eFigure 1. Illustrative Map of the United States of America Recording Expansion States as of December 2016 as Categorized by NCDB in Our Study

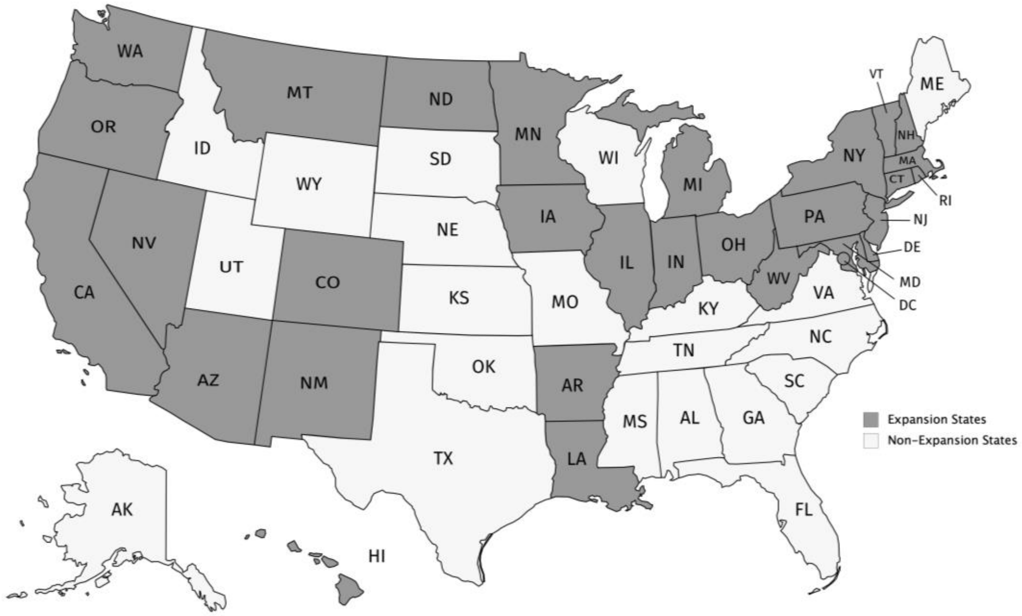

© 2020 Moyers JT et al. *JAMA Network Open.*

eFigure 2. CONSORT Diagram Listing Patient Allotment in Analysis

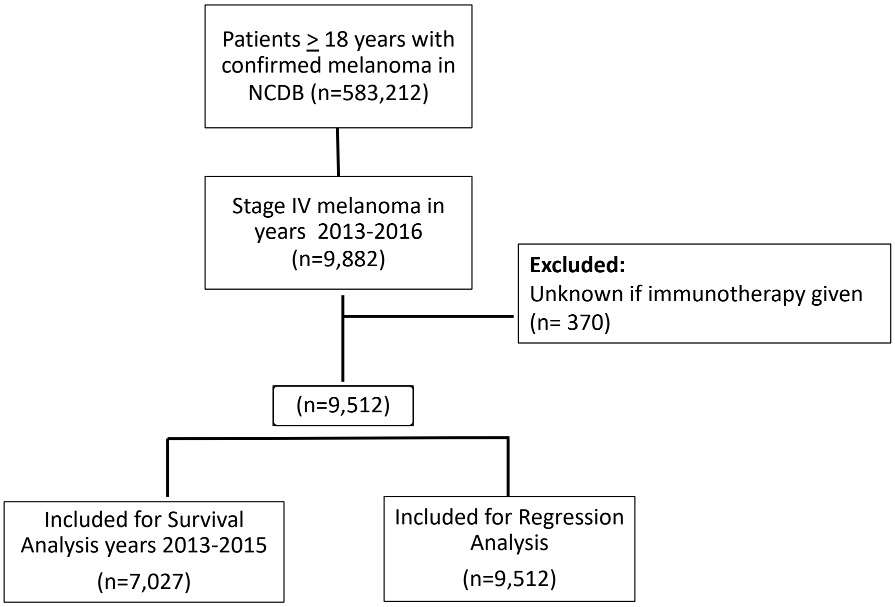

© 2020 Moyers JT et al. *JAMA Network Open.*

eFigure 3. Time From Diagnosis to Treatment With Immunotherapy in Days Separated by State Medicaid Expansion Status

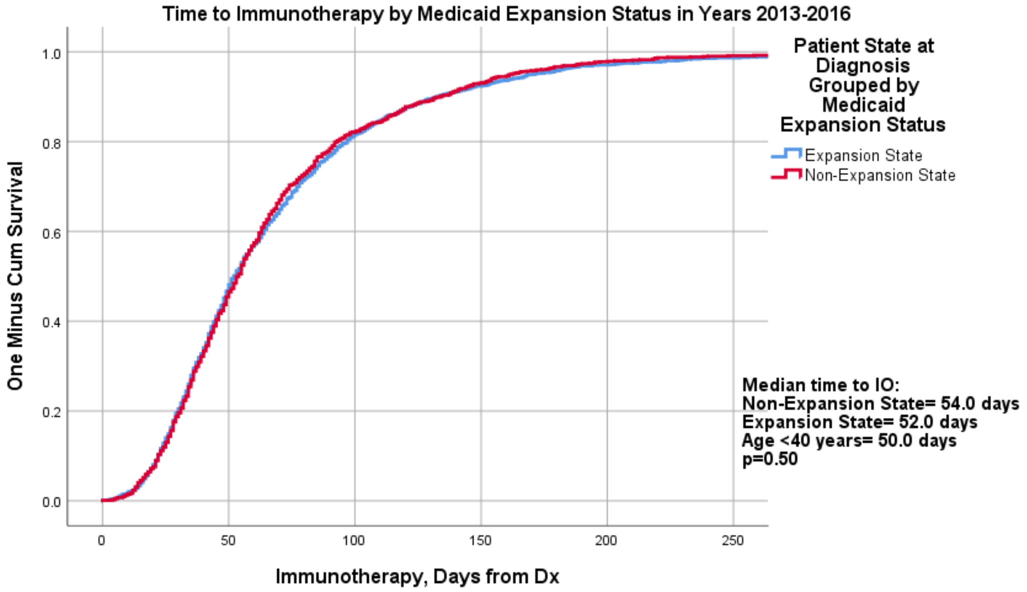

© 2020 Moyers JT et al. *JAMA Network Open.*

eFigure 4. Immunotherapy Receipt as Percentage of Total Population by Year

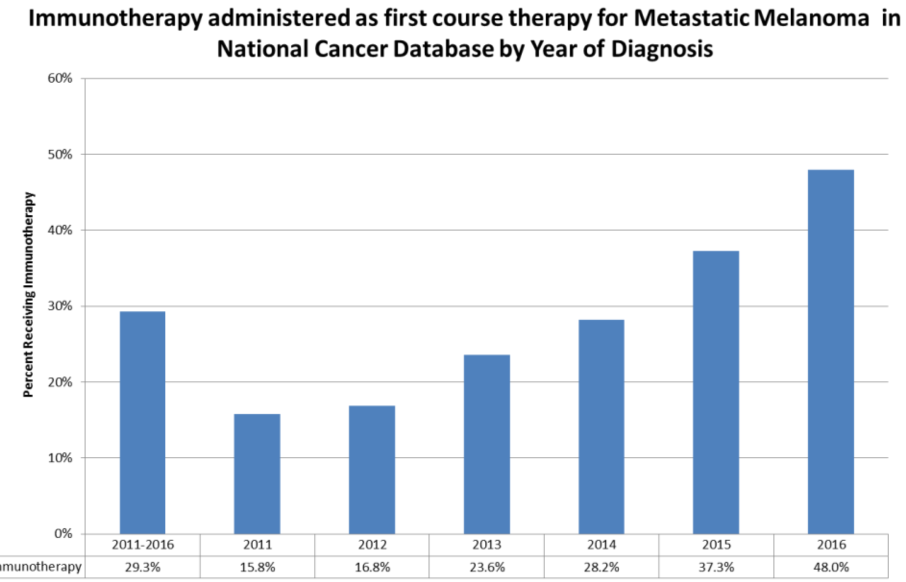

	2011-2016	2011	2012	2013	2014	2015	2016
Received Immunotherapy	29.3%	15.8%	16.8%	23.6%	28.2%	37.3%	48.0%

© 2020 Moyers JT et al. *JAMA Network Open.*

eTable. Payer Status at Time of Diagnosis by Medicaid Expansion Status

		Non-Expansion States (%)	Expansion States (%)	p-value
Total	8990	3571 (39.7%)	5419 (60.3%)	
Insurance Type				
Uninsured	329	204 (62.0%)	125 (38.0%)	<0.01
Private Insurance	3027	1133 (62.6%)	1894 (37.4%)	<0.01
Medicaid	602	179 (29.7%)	423 (70.3%)	<0.01
Medicare	4702	1871 (39.8%)	2831 (60.2%)	<0.01
Other Government	157	85 (54.1%)	72 (45.9%)	0.30
Unknown	173	99 (57.2%)	74 (42.8%)	0.06

© 2020 Moyers JT et al. *JAMA Network Open.*
